# Stigma towards people with mental disorders and its components – a perspective from multi-ethnic Singapore

**DOI:** 10.1017/S2045796016000159

**Published:** 2016-03-28

**Authors:** M. Subramaniam, E. Abdin, L. Picco, S. Pang, S. Shafie, J. A. Vaingankar, K. W. Kwok, K. Verma, S. A. Chong

**Affiliations:** 1 Research Division, Institute of Mental Health, Singapore; 2 Sociology Division, Nanyang Technological University, Singapore

**Keywords:** Alcohol abuse, Dementia, Depression, Mental illness stigma, Obsessive compulsive disorder, Schizophrenia

## Abstract

**Aims.:**

The current study aimed to: (i) describe the extent of overall stigma as well as the differences in stigma towards people with alcohol abuse, dementia, depression, schizophrenia and obsessive compulsive disorder, as well as (ii) establish the dimensions of stigma and examine its correlates, in the general population of Singapore, using a vignette approach.

**Methods.:**

Data for the current study came from a larger nation-wide cross-sectional study of mental health literacy conducted in Singapore. The study population comprised Singapore Residents (Singapore Citizens and Permanent Residents) aged 18–65 years who were living in Singapore at the time of the survey. All respondents were administered the Personal and Perceived scales of the Depression Stigma scale and the Social Distance scale to measure personal stigma and social distance, respectively. Weighted mean and standard error of the mean were calculated for continuous variables, and frequencies and percentages for categorical variables. Exploratory structural equation modelling and confirmatory factor analysis were used to establish the dimensions of stigma. Multivariable linear regressions were conducted to examine factors associated with each of the stigma scale scores.

**Results.:**

The mean age of the respondents was 40.9 years and gender was equally represented (50.9% were males). The findings from the factor analysis revealed that personal stigma formed two distinct dimensions comprising ‘weak-not-sick’ and ‘dangerous/unpredictable’ while social distance stigma items loaded strongly into a single factor. Those of Malay and Indian ethnicity, lower education, lower income status and those who were administered the depression and alcohol abuse vignette were significantly associated with higher weak-not-sick scores. Those of Indian ethnicity, 6 years of education and below, lower income status and those who were administered the alcohol abuse vignette were significantly associated with higher dangerous/unpredictable scores. Those administered the alcohol abuse vignette were associated with higher social distance scores.

**Conclusion.:**

This population-wide study found significant stigma towards people with mental illness and identified specific groups who have more stigmatising attitudes. The study also found that having a friend or family member with similar problems was associated with having lower personal as well as social distance stigma. There is a need for well-planned and culturally relevant anti-stigma campaigns in this population that take into consideration the findings of this study.

## Introduction

Stigma is defined by the World Health Organisation (WHO) as ‘a mark of shame, disgrace or disapproval that results in an individual being rejected, discriminated against and excluded from participating in a number of different areas of society’ (World Health Organization, 2001). Stigma has been linked to adverse outcomes for people with mental illness as it acts as a barrier to help-seeking as well as achievement of age-appropriate functional goals (Corrigan *et al.* 2009; Clement *et al.* 2015). In an attempt to circumvent the stigma associated with mental illness there is ‘label avoidance’ i.e. people are reluctant to be diagnosed with or be seen as seeking treatment for mental illness (Corrigan *et al.* 2014). Public stigma can also lead to ‘self-stigma’ (Link, 1987) among those with mental illnesses leading to shame, loss of self-esteem, withdrawal from academic or vocational pursuits (Corrigan & Watson, 2002; Corrigan *et al.* 2009), poor treatment adherence, increased symptom severity (Mak & Wu, 2006; Livingston & Boyd, 2010) and poor quality of life (Vauth *et al.* 2007).

Given that stigma is a social construct, culture impacts stigma significantly. Culture refers to the behaviours, beliefs, value orientations and symbols that a group of people have in common that influence their customs, norms and practices; and is socially transmitted across generations. These sociocultural norms and practices also determine the meaning, practice and expression of stigma across different populations (Yang *et al.* 2007; Cheon & Chiao, 2012). For example, cultural beliefs play a significant role in determining the explanatory models of illness (Kleinman, 1980) which in turn gives meaning to stigma. Abdullah & Brown (2011) in their review of the literature suggest that the ‘collectivist’ nature of Asians, leads to the perception that mental illnesses reflect flaws of the family. Supernatural attributions for mental illness are often viewed as a punishment for some individual or familial misdeed (Philips, 1993). Similarly, ‘bad deeds’ and ‘sins’ committed in the present or past lives may be perceived as a cause of the mental illness leading to the stigmatisation of those with these illnesses (Raguram *et al.* 2004). The inability of a person with mental illness to achieve academic and occupational successes that are highly regarded and valued in many cultures also leads to stigmatisation.

While the concept of stigma (and the stigmatisation) of those with mental illnesses has been studied widely in Western countries, relatively few studies have been carried out in Asian countries. The current study aims to bridge this gap by examining stigma among the adult population in an Asian society. Singapore is a multi-ethnic city state country in Southeast Asia, with a resident population of 3.8 million (Statistics Singapore, 2014) of which 74.2% are Chinese, 13.3% are Malays, 9.1% are Indians and 3.3% belong to other ethnic groups. Singapore has a robust developed economy and a highly literate population with English being the language of instruction in schools and government. However, culturally rooted traditions and beliefs specific to the various ethnic groups who have largely migrated from China, Malaysia, Indonesia and India are prevalent. An earlier study showed ethnic differences in the perception of mental health problems, with those of Malay ethnicity being the most tolerant of all the ethnic groups (Chong *et al.* 2007). More than one-third of those surveyed believed that those with mental disorders were dangerous and wanted to distance themselves from those with mental disorders. However, no study has since examined the extent or correlates of stigma towards mental illnesses at a population level.

The aims of the current study were to: (i) describe the extent of overall stigma as well as the differences in stigma towards people with alcohol abuse, dementia, depression, schizophrenia and obsessive compulsive disorder (OCD), as well as (ii) establish the dimensions of stigma and examine its correlates, in the general population of Singapore among those aged 18–65 years using a vignette approach.

## Methodology

### Sample

Data for the current study came from a larger nation-wide cross-sectional study of mental health literacy conducted in Singapore from March 2014 to April 2015. Statistical power calculations for binary proportions after adjusting for design effect were estimated to determine the sample size for the overall prevalence estimate, as well as for sub-groups by age and ethnicity, with precision of 4% (Kish, 1965). Sample size was derived using 20% as a prevalence estimate for correct recognition of causes of mental disorders in Singapore, as reported in an earlier study (Chong *et al.* 2007). A sample size of 600 was calculated for each vignette. A total sample size of 3000 (5 vignettes × 600 cases) with the margin of error was then computed and estimated to be adequate to provide sufficient precision for the study. We recalculated the adequacy of the sample size (i.e. *N* = 3000) for the stigma study using data from the study by Reavley & Jorm (2011), using prevalence estimate of respondents who ‘agree’ (2.5%) or ‘strongly agree’ (72.3%) with statements relating to personal stigma towards mental disorders. The target sample size of 3000 provided sufficient precision with the margin of error for the overall prevalence estimate found to be 0.08–2.2%, the margin of error for the strata defined by age and ethnicity to be 1.4–2.9% and relative standard error ranging from 1.5 to 26.7%, which was below the acceptable range of 30% (Klein *et al.* 2002).

The study population comprised Singapore Residents (Singapore Citizens and Permanent Residents) aged 18–65 years who were living in Singapore at the time of the survey. The sample was derived using the sampling frame from an administrative database in Singapore that maintains data on age, gender, ethnicity and residential address of all those residing in Singapore. Residents who were living outside the country and not contactable due to incomplete or incorrect addresses were excluded from the study. The study was approved by the relevant Institutional and Ethics Committees. Written informed consent was taken from all respondents who were 21 years and above as well as from parents or guardians of participants who were aged 18–20 years.

### Questionnaires

Mental health literacy was assessed using a questionnaire modelled on the Depression Literacy Questionnaire developed by Jorm *et al.* (1997). Respondents were randomly assigned and presented a vignette describing one of five specific disorders; alcohol abuse, dementia, depression, schizophrenia and OCD. While vignettes pertaining to depression and schizophrenia were adapted from those used in prior studies (Jorm *et al.* 1997; 2007), those pertaining to alcohol abuse, dementia, and OCD were developed by the investigators. All the vignettes were further revised in consultation with experienced research psychiatrists and vetted by a panel of senior clinical psychiatrists to ensure that these vignettes satisfied the Diagnostic and Statistical Manual of Mental Disorders 4th edition (DSM-IV) (American Psychiatric Association, 2000) diagnostic criteria. The case vignettes were further tested using cognitive interviews with 75 participants who were selected to represent different age-groups, genders, ethnicity and socio-economic strata. A clinician researcher (SAC) then vetted the final vignettes for equivalence across disorders by ensuring that the style of the vignette in terms of length, severity of the disorder and extent of non-essential details was consistent (Evans *et al.* 2015).

Each respondent was presented one vignette (predetermined by an algorithm) describing a person of the same gender and ethnicity as them. Sociodemographic information on all respondents was collected and included their age, gender, ethnicity, marital status, education, employment status and personal income. All respondents were administered the following two scales to measure stigma:

#### Personal and Perceived scales of the Depression Stigma Scale (DSS) (Griffiths *et al.* 2004)

The subscales each comprise nine items that address multiple facets of stigma by asking respondents about their own attitudes to the mentally ill person depicted in the vignette (personal stigma) and assessing the respondents beliefs about the attitudes of others to the person in the vignette (perceived stigma). While the scale was originally intended to measure depression stigma, it can also be administered in relation to vignettes of other disorders (Griffiths *et al.* 2006). For the purposes of this study only the eight-item DSS-personal subscale was used (‘I would not vote for a politician if I knew they had a mental illness’ item was not included).

#### Social Distance scale (SDS) (Link *et al.* 1999)

The scale measures self-reported willingness to make contact with the person described in the vignette. The scale score was calculated by summing item scores where higher scores indicate greater social distance.

The vignettes and the questionnaires were translated into the three local languages – Mandarin Chinese, Malay and Tamil by a professional translating firm. Administration of questionnaires was done in the language that the respondent was most familiar with.

### Statistical analyses

All estimates were weighted to adjust for over sampling and post-stratified for age and ethnicity distributions between the survey sample and the Singapore resident population in the year 2012. Weighted mean and standard error of the mean were calculated for continuous variables, and frequencies and percentages for categorical variables. To describe item endorsement, items on the personal stigma scale were recoded as three categories to indicate whether a participant – agrees; neither agrees nor disagrees and; disagrees – with these items ('agree’ and ‘strongly agree’; ‘disagree’ and ‘strongly disagree’ categories were combined) while items on the SDS were recoded as binary responses to indicate the percentage of participants willing/unwilling to interact with the person in the vignette (the ‘definitely unwilling’ and ‘probably unwilling’ categories were combined). On the basis of the extensive research evidence available in support for the underlying two factor structure of the personal stigma scale and one factor for SDS, we relied on a confirmatory approach to perform exploratory structural equation modelling (ESEM) for the estimation of a three-factor model and its comparison with an equivalent three-factor of the confirmatory factor analysis (CFA) solution (Yap *et al.* 2014; Amarasuriya *et al.* 2015). All structural equation modelling analyses were performed on polychoric correlation matrixes using Mplus version 7.0 with the weighted least squares with mean and variance adjusted chi-square statistic estimator for categorical indicators. CFA models were estimated according to the independent cluster model, with each item allowed to load on a single factor, and all factors allowed to correlate. ESEM models were estimated according to the specification provided in Asparouhov & Muthén (2009), with all rotated loadings freely estimated using an oblique Geomin rotation method. We also conducted separate multivariable linear regressions to examine factors associated with each of the stigma scale scores (continuous dependent variables) to examine which of the following dummy coded variables (independent variables) predicted the stigma scores: age, gender, ethnicity, marital status, education, employment status, income, type of vignette, if the problem in the vignette was experienced by family or friends and if the problem was experienced personally.

## RESULTS

Of the 4231 individuals contacted, 3006 respondents completed the study giving a response rate of 71%. Table 1 shows the sociodemographic characteristics of the respondents. The mean age of the respondents was 40.9 years. About 50.9% of the respondents were males and the majority were Chinese (74.7%). The random assignment of participants to vignette resulted in equivalent groups across vignettes in terms of gender, income, education, age and marital status; chi-square analysis revealed that no significant differences were found in sociodemographic variables across vignettes groups. Table 2a shows the endorsement of items on the personal stigma scale and SDS by the respondents. Endorsement of items by vignette is shown in Table 2b.

**Table 1.** Sociodemographic characteristics of the study sample

	*N*	Weighted %	s.e.
Age group			
18–34 years	1152	34.4	0.04
35–49 years	896	35.2	0.04
50–65 years	958	30.4	0.1
Gender			
Female	1506	49.1	1.3
Male	1500	50.9	1.3
Ethnicity			
Chinese	1034	74.7	0.04
Malay	977	12.8	0.01
Indian	963	9.1	0.01
Others	32	3.3	0.04
Marital status			
Married	1916	64.0	1.0
Never married	927	31.4	0.9
Others (divorced, widowed, separated)	162	4.6	0.5
Education			
Primary education and below	431	13.4	0.8
Secondary education include O/N level	820	25.7	1.0
A level, polytechnic and other diploma	999	31.3	1.1
University	756	29.6	1.1
Employment status			
Employed	2227	77.6	1.0
Housewife/homemaker	378	8.7	0.6
Retired	78	3.0	0.4
Student	203	6.7	0.5
Unemployed	120	3.9	0.5
Income			
<SGD2000	1346	40.5	1.2
SGD2000–5999	1162	46.4	1.3
SGD6000 and above	294	13.1	0.9
Vignette type			
Alcohol abuse	603	20.8	1.0
Dementia	596	19.2	1.0
Depression	607	19.9	1.0
OCD	605	20.5	1.0
Schizophrenia	595	19.6	1.0
*Ever had problems similar to person described in vignette*	319	11.4	0.8
*Family or close circle of friends ever had problems similar to person described in vignette*	700	22.6	1.1

Note: The weighted prevalence estimates may not sum to 100 due to rounding.

**Table 2a.** Item endorsement of the Depression Stigma Scale – personal stigma and social distance scale

Item	*n*	%	*n*	%	*n*	%
Personal stigma	Disagree		Neither agree nor disagree		Agree	
DSS – PS1 People with a problem like [insert male/female name]'s could get better if they wanted to	145	4.8	174	5.8	2687	89.4
DSS – PS2 A problem like [insert male/female name]'s is a sign of personal weakness	869	29.0	608	20.2	1525	50.8
DSS – PS3 [insert male/female name]'s problem is not a real medical illness	1355	45.2	488	16.3	1153	38.5
DSS – PS4 People with a problem like [insert male/female name]'s are dangerous to others	1323	44.0	611	20.3	1072	35.7
DSS – PS5 It is best to avoid people with a problem like [insert male/female name]'s so that you don't also get this problem	2327	77.4	361	12.0	318	10.6
DSS – PS6 People with a problem like [insert male/female name]'s are unpredictable	585	19.5	543	18.1	1877	62.5
DSS – PS7 If I had a problem like [insert male/female name]'s I would not tell anyone	1953	65.2	432	14.4	611	20.4
DSS – PS8 I would not employ someone if I knew they had a problem like [insert male/female name]'s	899	30.0	742	24.7	1357	45.3
Social distance	Willing		Unwilling			
SD-1 How willing would you be to move next door to [insert male/female name]?	2027	67.6	973	32.4	–	–
SD-2 How willing would you be to spend an evening with [insert male/female name]?	2329	77.6	671	22.4	–	–
SD-3 How willing would you be to make friends with [insert male/female name]?	2451	81.8	544	18.2	–	–
SD-4 How willing would you be to have [insert male/female name] start working closely with you on a job?	1713	57.2	1283	42.8	–	–
SD-5 How willing would you be to have [insert male/female name] marry into your family?	886	29.8	2085	70.2	.

**Table 2b.** Item endorsement of the Depression Stigma Scale – personal stigma and social distance scale by vignette

	Alcohol abuse			Dementia			Depression			Schizophrenia			OCD		
Item	*n* (%)	*n* (%)	*n* (%)	*n* (%)	*n* (%)	*n* (%)	*n* (%)	*n* (%)	*n* (%)	*n* (%)	*n* (%)	*n* (%)	*n* (%)	*n* (%)	*n* (%)
Personal stigma	Disagree	Neither agree nor disagree	Agree	Disagree	Neither agree nor disagree	Agree	Disagree	Neither agree nor disagree	Agree	Disagree	Neither agree nor disagree	Agree	Disagree	Neither agree nor disagree	Agree
PS1	12 (1.9)	22.2 (3.5)	592 (94.5)	56 (9.6)	53 (9.1)	470 (81.3)	21 (3.5)	26 (4.3)	551 (92.1)	32 (5.4)	39 (6.6)	517 (88)	25 (4)	34 (5.6)	556 (90.4)
PS2	113 (18.2)	141 (22.7)	369 (59.1)	290 (50.1)	91 (15.7)	197 (34.1)	154 (25.7)	98 (16.4)	347 (57.9)	145 (24.7)	140 (23.8)	302 (51.5)	167 (27.2)	138 (22.4)	310 (50.4)
PS3	227 (36.3)	80 (12.7)	319 (51)	336 (58.4)	88 (15.3)	151 (26.2)	242 (40.7)	111 (18.7)	241 (40.6)	279 (47.8)	120 (20.6)	185 (31.7)	270 (43.9)	89 (14.5)	256 (41.6)
PS4	116 (18.5)	103 (16.4)	407 (65.1)	284 (49.2)	101 (17.4)	193 (33.4)	330 (55.1)	136 (22.7)	132 (22.1)	136 (23.2)	189 (32.1)	263 (44.7)	456 (74.2)	83 (13.4)	76 (12.4)
PS5	367 (58.6)	122 (19.5)	137 (21.9)	509 (88.1)	34 (5.9)	34 (5.9)	517 (86.3)	57 (9.5)	25 (4.2)	399 (68)	102 (17.3)	87 (14.8)	535 (86.9)	46 (7.4)	35 (5.6)
PS6	106 (17)	113 (18)	407 (65)	82 (14.2)	89 (15.5)	406 (70.3)	119 (19.9)	117 (19.5)	362 (60.6)	58 (9.8)	77 (13.2)	452 (77)	220 (35.7)	146 (23.8)	249 (40.5)
PS7	372 (59.8)	82 (13.2)	169 (27.1)	434 (75.6)	71 (12.4)	69 (12)	415 (69.6)	88 (14.8)	92 (15.5)	344 (58.6)	88 (15)	155 (26.3)	387 (62.9)	102 (16.5)	126 (20.5)
PS8	135 (21.6)	103 (16.5)	387 (61.8)	149 (25.9)	151 (26.2)	275 (47.9)	221 (37.1)	189 (31.8)	185 (31.1)	114 (19.4)	157 (26.7)	317 (54)	280 (45.6)	142 (23.1)	192 (31.3)
Social distance	Willing	Unwilling		Willing	Unwilling			Willing	Unwilling	Willing	Unwilling		Willing	Unwilling	
SD1	306 (48.8)	320 (51.2)		476 (82.4)	102 (17.6)			480 (80.8)	114 (19.2)	270 (45.9)	318 (54.1)		496 (80.6)	119 (19.4)	
SD2	441 (70.5)	185 (29.5)		498 (86.1)	80 (13.9)			508 (85.2)	88 (14.8)	360 (61.4)	226 (38.6)		522 (85.1)	91 (14.9)	
SD3	459 (73.4)	167 (26.6)		531 (91.8)	47 (8.2)			523 (88.4)	69 (11.6)	386 (66)	199 (34)		552 (89.8)	63 (10.2)	
SD4	300 (48.1)	323 (51.9)		311 (53.8)	267 (46.2)			419 (70.4)	176 (29.6)	259 (44.2)	327 (55.8)		425 (69.1)	190 (30.9)	
SD5	106 (16.9)	520 (83.1)		180 (31.3)	396 (68.7)			242 (41.4)	343 (58.6)	113 (19.4)	467 (80.6)		245 (40.5)	360 (59.5)	

Note: PS1 = DSS – PS1 People with a problem like [insert male/female name]'s could get better if they wanted to. PS2 = DSS – PS2 A problem like [insert male/female name]'s is a sign of personal weakness. PS3 = DSS – PS3 [insert male/female name]'s problem is not a real medical illness. PS4 = DSS – PS4 People with a problem like [insert male/female name]'s are dangerous to others; PS5 = DSS – PS5 It is best to avoid people with a problem like [insert male/female name]'s so that you don't also get this problem. PS6 = DSS – PS6 People with a problem like [insert male/female name]'s are unpredictable. PS7 = DSS – PS7 If I had a problem like [insert male/female name]'s I would not tell anyone. PS8 = DSS – PS8 I would not employ someone if I knew they had a problem like [insert male/female name]'s. SD1 = SD-1 How willing would you be to move next door to [insert male/female name]? SD2 = SD-2 How willing would you be to spend an evening with [insert male/female name]? SD3 = SD-3 How willing would you be to make friends with [insert male/female name]? SD4 = SD-4 How willing would you be to have [insert male/female name] start working closely with you on a job? SD5 = SD-5 How willing would you be to have [insert male/female name] marry into your family?

The table providing the factor loadings and model fit for the CFA and ESEM models of the personal stigma scale and SDS is available online as Supplementary material (Table 1). The three factors based on ESEM geomin rotation solution (model 3) provided an acceptable fit. Although this model indicated a good fit, the factor loading for the item ‘if I had problem like the subject's I would not tell anyone’ was very poor. Therefore, we decided to exclude this item and rerun the model (ESEM model 4). This model improved and fit well, with acceptable factor loadings.

The findings from the factor analysis revealed that personal stigma formed two distinct dimensions comprising ‘weak-not-sick’ and ‘dangerous/unpredictable’, similar to that reported by Yap *et al.* (2014). The factor labelled as ‘weak-not-sick’ was defined by three items (DSS – PS1, DSS – PS2 and DSS – PS3) which characterise the problem portrayed in the vignette as a personal weakness, under the control of the person rather than as a medical condition. The factor labelled ‘dangerous/unpredictable’ was defined by four items (DSS – PS4, DSS – PS5, DSS – PS6 and DSS – PS8) and included those perceiving the person as dangerous, unpredictable and as someone best avoided. The item concerning not employing this person also loaded into this factor. The social distance stigma items loaded strongly into a single factor (SD –1 to SD –5).

Table 3 reports the descriptive values of ‘weak-not-sick’, ‘dangerous/unpredictable’ and ‘social distance’ stigma dimensions across sociodemographic groups. Table 4 shows the correlates of the three stigmatising attitudes factor scores calculated by summing items with substantial loadings (>0.30) derived from the ESEM Model 4 (higher scores reflect higher level of stigma). Multivariable linear regressions analyses revealed that age, gender, ethnicity, education, vignette type and those who endorsed that a family member or close friend ever had problems similar to the person in the vignette were significantly and consistently associated with all three factors.

**Table 3.** Descriptive statistics of stigma dimension scores by sociodemographic factors

	Weak-not-sick			Dangerous-undesirable			Social distance		
	Mean	s.e.	*p* value	Mean	s.e.	*p* value	Mean	s.e.	*p* value
Age group									
18–34 years	9.58	0.08	<0.001	10.88	0.10	<0.001	11.58	0.10	0.0024
35–49 years	10.30	0.10		11.63	0.12		11.71	0.12	
50–65 years	10.92	0.08		12.43	0.12		12.18	0.14	
Gender									
Female	10.23	0.08	0.7701	11.53	0.09	0.0744	11.49	0.10	<0.001
Male	10.26	0.07		11.69	0.10		12.11	0.10	
Ethnicity									
Chinese	10.07	0.06	<0.001	11.61	0.08	0.6626	12.00	0.09	<0.001
Others	9.91	0.48		11.42	0.52		11.71	0.45	
Malay	10.95	0.06		11.60	0.09		10.89	0.09	
Indian	10.74	0.08		11.75	0.11		11.52	0.11	
Marital status									
Married	10.48	0.07	<0.001	11.81	0.08	<0.001	11.83	0.09	0.4527
Others (divorced, widowed, separated)	10.67	0.28		12.37	0.27		12.15	0.38	
Never married	9.70	0.09		11.11	0.11		11.71	0.11	
Education									
Primary education and below	11.05	0.11	<0.001	12.66	0.18	<0.001	12.10	0.22	0.0099
Secondary education include O/N level	10.87	0.09		11.90	0.13		11.48	0.15	
A level, polytechnic and other diploma	10.24	0.09		11.30	0.12		11.71	0.11	
University	9.33	0.10		11.22	0.12		12.06	0.13	
Employment status									
Employed	10.24	0.06	<0.001	11.61	0.08	<0.001	11.83	0.08	0.227
Housewife/homemaker	10.91	0.15		12.17	0.18		11.53	0.25	
Retired	10.96	0.29		12.99	0.38		12.47	0.35	
Student	9.16	0.18		10.50	0.24		11.62	0.22	
Unemployed	9.97	0.25		11.30	0.33		11.78	0.41	
Income									
<SGD2000	10.49	0.08	<0.001	11.77	0.10	0.1917	11.64	0.11	0.0054
SGD2000–5999	10.13	0.08		11.55	0.10		11.83	0.10	
SGD6000 and above	9.72	0.16		11.43	0.19		12.39	0.20	
Vignette type									
Alcohol abuse	10.93	0.11	<0.001	13.20	0.14	<0.001	12.88	0.15	<0.001
Dementia	9.23	0.12		11.47	0.13		11.34	0.13	
Depression	10.55	0.12		10.80	0.12		10.85	0.13	
OCD	10.28	0.11		9.90	0.13		10.89	0.13	
Schizophrenia	10.14	0.11		12.69	0.13		13.05	0.17	
Ever had problems similar to person described in vignette									
No	10.23	0.05	0.5396	11.73	0.07	<0.001	11.93	0.07	<0.001
Yes	10.34	0.18		10.72	0.18		10.87	0.20	
Family or close circle of friends ever had problems similar to person described in vignette									
No	10.25	0.06	0.8841	11.67	0.07	0.1033	11.97	0.08	<0.001
*Yes*	10.23	0.11		11.42	0.14		11.25	0.14

**Table 4.** Multivariate linear regression analyses for variables predicting stigma dimensions

	Weak-not-sick				Dangerous-undesirable				Social distance			
	*β*	95% CI		*P* value	*β*	95% CI		*p* value	*β*	95% CI		*P* value
Age group												
18–34 years	−0.442	−0.683	−0.201	0.003	−0.896	−1.2	−0.592	<0.001	−0.639	−0.968	−0.31	0.001
35–49 years	−0.027	−0.226	0.173	0.792	−0.333	−0.605	−0.061	0.016	−0.219	−0.514	0.077	0.147
50–65 years	Ref*				Ref				Ref			
Gender												
Female	−0.175	−0.335	−0.016	0.031	−0.352	−0.559	−0.145	0.001	−0.48	−0.696	−0.265	<0.001
Male	Ref				Ref				Ref			
Ethnicity												
Others	0.115	−0.616	0.845	0.758	−0.16	−0.847	0.528	0.649	−0.324	−1.231	0.582	0.483
Malay	0.615	0.435	0.795	<0.001	−0.12	−0.355	0.115	0.316	−1.1	−1.344	−0.857	<0.001
Indian	0.728	0.538	0.919	<0.001	0.255	0.014	0.496	0.038	−0.448	−0.711	−0.184	0.001
Chinese	Ref				Ref				Ref			
Marital status												
Others (divorced, widowed, separated)	0.023	−0.312	0.359	0.892	0.069	−0.398	0.535	0.773	0.168	−0.333	0.669	0.511
Never married	−0.549	−0.758	−0.339	<0.001	−0.255	−0.517	0.006	0.056	0.016	−0.27	0.302	0.911
Married	Ref				Ref				Ref			
Education												
Primary education and below	1.209	0.884	1.534	<0.001	0.911	0.488	1.334	<0.001	−0.416	−0.871	0.039	0.073
Secondary education include O/N level	1.118	0.858	1.379	<0.001	0.201	−0.113	0.515	0.210	−0.579	−0.912	−0.247	0.001
A level, polytechnic and other diploma	0.753	0.523	0.982	<0.001	0.142	−0.127	0.41	0.301	−0.209	−0.49	0.072	0.144
University	Ref				Ref				Ref			
Employment status												
Housewife/homemaker	0.097	−0.194	0.388	0.513	0.046	−0.338	0.43	0.815	0.03	−0.387	0.447	0.888
Retired	−0.303	−0.829	0.223	0.259	0.209	−0.52	0.938	0.575	0.481	−0.34	1.301	0.251
Student	−0.611	−0.967	−0.255	0.001	−0.482	−0.943	−0.021	0.041	−0.056	−0.487	0.376	0.800
Unemployed	−0.266	−0.683	0.152	0.212	−0.069	−0.638	0.499	0.811	0.041	−0.603	0.685	0.901
Employed	Ref				Ref				Ref			
Income												
<SGD2000	0.565	0.236	0.894	0.001	0.676	0.28	1.072	0.001	0.311	−0.121	0.744	0.159
SGD2000–5999	0.306	0.012	0.601	0.041	0.536	0.194	0.878	0.002	0.145	−0.23	0.52	0.449
SGD6000 and above	Ref				Ref				Ref			
Vignette type												
Alcohol abuse	1.034	0.802	1.267	<0.001	0.943	0.634	1.252	<0.001	0.429	0.081	0.776	0.016
Dementia	−0.600	−0.838	−0.361	<0.001	−1.086	−1.38	−0.792	<0.001	−1.418	−1.738	−1.098	<0.001
Depression	0.345	0.109	0.582	0.004	−1.507	−1.813	−1.202	<0.001	−1.775	−2.122	−1.428	<0.001
OCD	0.200	−0.038	0.438	0.100	−2.547	−2.849	−2.244	<0.001	−2.214	−2.538	−1.89	<0.001
Schizophrenia	Ref				Ref				Ref			
*Ever had problems similar to person described in vignette*	0.187	−0.076	0.451	0.163	−0.58	−0.915	−0.245	0.001	−0.591	−0.937	−0.246	0.001
*Family or close circle of friends ever had problems similar to person described in vignette*	−0.216	−0.4	−0.032	0.022	−0.284	−0.531	−0.038	0.024	−0.515	−0.766	−0.263	<0.001

Ref*, reference category.

The factor correlation and scale reliabilities are included in the Supplementary material as Table 2. The correlations between the factors were not very strong (though significant) with dangerous-unpredictable showing a significant positive correlation with social distance.

## Discussion

The results of this study revealed that there is considerable personal stigma towards mental illness. Those who received the alcohol abuse vignette endorsed more stigmatising attitudes, compared with the other four vignettes, with the exception of the item relating to unpredictability, where schizophrenia was found to have the most stigmatising endorsement. Interestingly in terms of social distance, those who received the schizophrenia vignette endorsed the highest ‘unwillingness’ on all items of the scale except one (SD5) wherein those receiving the alcohol abuse vignette endorsed that they were most unwilling for the person with the problems to be married into their family.

The ESEM analysis revealed that the personal stigma scale comprised two distinct components – ‘weak-not-sick’ and ‘dangerous/unpredictable’, similar to other studies (Yap *et al.* 2011, 2014). The reluctance to disclose item – ‘If I had a problem like the person in the vignette, I would not tell anyone’ loaded on all three stigma dimensions. The desire to conceal mental illness can be considered a proxy for anticipatory discrimination which stems from an awareness of negative perception and discrimination towards those with mental illness. Anticipatory discrimination may also be a result of perceived social distancing towards those with mental illness, however, those with anticipatory discrimination may also tend to socially isolate themselves due to loss of confidence and self-esteem (Farrelly *et al.* 2014). The desire to conceal is thus a complex construct that may be associated with all three dimensions of stigma. On the other hand, the SDS measured a single distinct dimension similar to that reported across several studies (Yap *et al.* 2014; Yoshioka *et al.* 2014; Amarasuriya *et al.* 2015) attesting to its cross-cultural applicability.

Largely similar correlates were identified across all three dimensions of stigma. Those belonging to the younger age group, female gender, and those who endorsed that a family member or close friend ever had problems similar to the person in the vignette were significantly associated with lower scores on all three dimensions. Previous studies, including one conducted locally, found that younger people were more tolerant and less stigmatising (Chong *et al.* 2007; Griffiths *et al.* 2008; Reavley *et al.* 2014). This may be a reflection of changing knowledge and perceptions about mental illness. It could also be due to the fact that younger people are better informed about the causes, treatment and outcomes of mental illness as a result of exposure to campaigns in places of education, as well as through social media. A recent study by Schomerus *et al.* (2015) found that social distance increased with age and that this effect was independent of cohort effect. The authors suggested that their findings may reflect the increasing conservatism associated with growing age or it may be due to active preference of positive contacts and relationships by older adults in accordance with the socio-emotional selectivity theory (Carstensen *et al.* 1999). A few studies have found gender differences similar to that seen in our study (Corrigan & Watson, 2007; Reavley *et al.* 2014; Yap *et al.* 2014). As reported in previous studies (Lauber *et al.* 2004; Crisp *et al.* 2005; Griffiths *et al.* 2008), exposure to mental illness was associated with lower personal stigma and lower social distance. It may be more likely that those with personal contact with a person with mental illness have a better understanding of mental illness. It is also possible that they are more sensitive to stigma and discrimination against people with mental illness. Lastly, if they have family members with mental illness it may be significantly more likely that the respondents themselves have been subjected to stigmatisation leading to empathy and non-stigmatising attitudes. Social contact with people with mental illness was also construed to be an effective anti-stigma intervention (Corrigan *et al.* 2012). An evaluation of mass social contact events in the Time to Change (TTC) campaign which facilitate positive contact between those with and without mental illness concluded that the interaction was associated with improved behavioural intentions, i.e. increased intent to have contact with someone with mental illness (Evans-Lacko *et al.* 2012).

Those with lower education and lower income were significantly associated with higher scores on the personal stigma scale. This finding too has been reported by other studies (Corrigan & Watson, 2007; Griffiths *et al.* 2008) and it is suggested that those with higher education have more knowledge and thus a better understanding about people with mental illness. Our findings overall, are thus largely similar to studies from other cultural settings and suggest that the characteristics of mental illness stigma, in terms of concept and correlates are stronger than cultural differences.

Significant ethnic differences were observed in mental illness stigma. Those of Malay ethnicity were significantly associated with higher weak-not-sick scores and with lower social distance scores. It is possible that while the Malays perceive mental illness as a weakness and not a real illness, they are more tolerant and accepting of people with such symptoms and thus do not segregate them. This tolerance could be due to their cultural and religious values. Most Malays in Singapore follow Islam and according to the tenets of Islam, mental illness is perceived as a test from God (Abu-Ras & Abu-Bader, 2008; Rassool, 2000). Illness may also be seen as an opportunity to reconnect and resolve their lack of faith through regular prayer and a sense of self-responsibility (Youssef & Deane, 2006; Padela *et al.* 2012). Those of Indian ethnicity, on the other hand, were significantly associated with higher weak-not-sick and dangerous/unpredictable scores; however, they were associated with lower scores on social distance. We are unable to explain this finding, as it seems to suggest that while people with mental illness are perceived as dangerous/unpredictable, Indians are more willing to include them in the community and as part of their lives. Anglin *et al.* (2006) reported similar findings while examining racial differences in stigmatising attitudes in the USA. Their study found that while African Americans were more likely to believe that people with mental illness would be violent, they were less likely to blame them and were more accommodating towards them. Previous studies suggest that the prevalence of both depression and alcohol use disorder is higher among those of Indian ethnicity (Chong *et al.* 2012; Subramaniam *et al.* 2012) in Singapore and it is thus interesting to postulate an association between higher prevalence and lower social distancing within a community. However, other studies have concluded that generally estimates of prevalence were not related to a desire for social distance (Von dem Knesebeck *et al.* 2013); more research is therefore needed to understand this association in the local context. Qualitative studies which can provide a more in-depth understanding of the phenomenon are needed to better understand these ethnic differences in stigma.

Those administered the depression vignette were more likely to perceive the person described in it as weak-not-sick as compared with those administered the schizophrenia vignette. This may be tied into people's perception of depression as having a psychosocial aetiology rather than a biological aetiology thereby leading to the assumption that one can be resilient and not succumb to it. A study from Italy (Munizza *et al.* 2013) on public beliefs and attitudes towards depression similarly found that the respondents felt that someone with depression could get better ‘with some willpower’. Dementia, depression and OCD were associated with lower dangerous/unpredictable as well as social distance scores as compared with schizophrenia. Alcoholism on the other hand was perceived as even more dangerous/unpredictable than schizophrenia. Schomerus *et al.* (2011) observed a clustering effect similar to our study, with alcohol addiction and schizophrenia at one end of the spectrum and depression, anxiety disorders and dementia at the other end with the latter being perceived as less dangerous.

In the current study, the alcohol abuse vignette was associated with the highest stigma, i.e. respondents were significantly more likely to perceive them as weak-not-sick, dangerous/unpredictable and express the need for greater social distancing. Previous research comparing stigma of other mental illnesses to substance abuse have similarly found that people who abuse substances were perceived as more responsible for their disorder and more dangerous (Link *et al.* 1999; Corrigan & Watson, 2007). Schomerus *et al.* (2011) found that alcoholism was less likely to be perceived as a mental disorder, and those with alcoholism were considered to be more dangerous and the desire for social distance towards them was stronger. The authors suggested that while schizophrenia may be identified as an illness for which an individual does not bear any responsibility, alcoholism may be perceived as voluntary and therefore not a mental illness. Phelan *et al.* (2008) suggested that ‘voluntary’ behaviours like substance-related addictions may be stigmatised to reinforce social norms and conformity.

The strengths of this study include the large sample size, good response rate and the comparison across multiple illnesses using standardised questionnaires. The limitations include the cross-sectional design of the study which precludes any causal inferences. The reliance on self-report by the respondents carries a higher risk of social desirability bias, especially since the questions measured stigmatising attitudes. While the response rate was 71%, the fact remains that about 29% of those approached did not participate in the survey. It is possible that this group could have held very different views as compared with those who participated in the survey. It is possible that the scales used in the study (DSS and SDS) though having similar factor structures to that observed in other populations may not be measuring same constructs; the investigators had conducted cognitive interviews to confirm the understanding of the items of the scale and found that respondents understood the scale and the construct it was measuring in the way it was intended. However, there may be other aspects of stigma or social distancing relevant to this multi-ethnic population that were not included in these scales. The study would also have been further strengthened by including an instrument that measures behavioural discrimination such as the Reported and Intended Behaviour scale (Evans-Lacko *et al.* 2011). Lastly, the use of vignettes has some inherent limitations as a respondent's behaviour in response to a hypothetical scenario may differ considerably from that in real life. However, the previous research has demonstrated that that there is significant concordance between hypothetical and actual behaviours (Evans *et al.* 2015).

In conclusion, this population-wide study found significant stigma towards mental illness and identified specific groups who have more stigmatising attitudes. Thus, there is a need for well-planned and culturally relevant anti-stigma campaigns in this population that take into consideration the findings of this study. Groups endorsing higher stigmatising attitudes such as those who are older, males and less educated should be engaged and campaigns targeted towards them. Social contact with people with mental illness which has a role in reducing stigma must be incorporated in these campaigns.

## Supplementary material

For supplementary material accompanying this paper visit http://dx.doi.org/10.1017/S2045796016000159. click here to view supplementary material
