# Supplementary material for: Stigma towards people with mental disorders and its components – a perspective from multi-ethnic Singapore
Source: Epidemiol Psychiatr Sci. 2016 Mar 28;26(4):371–82. doi: 10.1017/S2045796016000159 (PMC5647661; doi:10.1017/S2045796016000159)
Supplement: Supplementary file 1 [file S2045796016000159sup001.docx]

**Supplementary Material**

**Table 1 Factor loadings and model fits for the CFA and ESEM models of the Personal Stigma and Social Distance questionnaires**

|  | **CFA** | | | **ESEM** | | | | | | | | |
| --- | --- | --- | --- | --- | --- | --- | --- | --- | --- | --- | --- | --- |
|  | **Model 1** | | | **Model 2** | | | **Model 3** | | | **Model 4** | | |
|  | Factor 1 | Factor 2 | Factor 3 | Factor 1 | Factor 2 | Factor 3 | Factor 1 | Factor 2 | Factor 3 | Factor 1 | Factor 2 | Factor 3 |
| DSS –PS1 People with a problem like [insert male/female name]'s could get better if they wanted to | 0.28 |  |  | 0.36 | -0.12 |  | 0.31 | 0.01 | -0.09 | 0.32 | 0.01 | -0.08 |
| DSS –PS2 A problem like [insert male/female name]'s is a sign of personal weakness | 0.89 |  |  | 0.81 | 0.01 |  | 0.76 | 0.07 | 0.01 | 0.79 | 0.07 | 0.03 |
| DSS –PS3 [insert male/female name]'s problem is not a real medical illness | 0.55 |  |  | 0.53 | 0.05 |  | 0.60 | -0.02 | 0.10 | 0.57 | -0.01 | 0.09 |
| DSS –PS4 People with a problem like [insert male/female name]'s are dangerous to others. |  | 0.59 |  | 0.20 | 0.53 |  | 0.14 | 0.70 | -0.06 | 0.14 | 0.71 | -0.06 |
| DSS –PS5 It is best to avoid people with a problem like [insert male/female name]’s so that you don't also get this problem. |  | 0.68 |  | 0.26 | 0.61 |  | 0.28 | 0.55 | 0.12 | 0.27 | 0.54 | 0.13 |
| DSS –PS6 People with a problem like [insert male/female name]'s are unpredictable |  | 0.39 |  | 0.13 | 0.34 |  | 0.05 | 0.52 | -0.12 | 0.05 | 0.52 | -0.11 |
| DSS –PS7 If I had a problem like [insert male/female name]'s I would not tell anyone |  | 0.33 |  | 0.03 | 0.33 |  | 0.12 | 0.15 | 0.18 |  |  |  |
| DSS –PS8 I would not employ someone if I knew they had a problem like [insert male/female name]'s. |  | 0.68 |  | -0.13 | 0.72 |  | -0.06 | 0.48 | 0.25 | -0.07 | 0.46 | 0.25 |
| SD-1 How willing would you be to move next door to [insert male/female name]? |  |  | 0.81 |  |  | 0.81 | -0.01 | 0.23 | 0.68 | -0.01 | 0.23 | 0.68 |
| SD-2 How willing would you be to spend an evening with [insert male/female name]? |  |  | 0.88 |  |  | 0.88 | 0.07 | 0.03 | 0.89 | 0.07 | 0.03 | 0.89 |
| SD-3 How willing would you be to make friends with [insert male/female name]? |  |  | 0.91 |  |  | 0.91 | 0.03 | 0.05 | 0.90 | 0.03 | 0.05 | 0.90 |
| SD-4 How willing would you be to have [insert male/female name] start working closely with you on a job? |  |  | 0.71 |  |  | 0.71 | -0.24 | 0.29 | 0.55 | -0.24 | 0.28 | 0.56 |
| SD-5 How willing would you be to have [insert male/female name] marry into your family? |  |  | 0.69 |  |  | 0.69 | -0.12 | 0.40 | 0.44 | -0.12 | 0.39 | 0.45 |
| Fit indices |  |  |  |  |  |  |  |  |  |  |  |  |
| CFI (0.95) |  |  |  | 0.941 |  | 0.937 |  |  | 0.96 |  |  | 0.974 |
| TLI (0.75) |  |  |  | 0.955 |  | 0.957 |  |  | 0.965 |  |  | 0.976 |
| RMSEA (0.08) |  |  |  | 0.077 |  | 0.075 |  |  | 0.067 |  |  | 0.06 |
| χ^2^ |  |  |  | 654.799 |  | 700.587 |  |  | 449.194 |  |  | 306.289 |
| Degree freedom |  |  |  | 35 |  | 39 |  |  | 31 |  |  | 26 |

**Table 2 Factor Correlations and Scale Reliability**

|  | Spearman correlation between factor scores | | | Reliability |
| --- | --- | --- | --- | --- |
|  | Weak-not-Sick | Dangerous-Undesirable | Social Distance | Cronbach’s alpha |
| Weak-not-Sick | - | 0.214** | -0.014 | 0.55 |
| Dangerous-Undesirable | 0.214** | - | 0.453** | 0.66 |
| Social Distance | -0.014 | 0.453** | - | 0.85 |

** p value < 0.001.
